# Optimal contouring of seminal vesicle for definitive radiotherapy of localized prostate cancer: comparison between EORTC prostate cancer radiotherapy guideline, RTOG0815 protocol and actual anatomy

**DOI:** 10.1186/s13014-014-0288-1

**Published:** 2014-12-20

**Authors:** Xin Qi, Xian-Shu Gao, Junichi Asaumi, Min Zhang, Hong-Zhen Li, Ming-Wei Ma, Bo Zhao, Fei-Yu Li, Dian Wang

**Affiliations:** Department of Radiation Oncology, Peking University First Hospital, Beijing, China; Department of Oral and Maxillofacial Radiology, Field of Tumor Biology, Okayama University Graduate School of Medicine, Dentistry and Pharmaceutical Sciences, Okayama, Japan; Department of Radiology, Peking University First Hospital, Beijing, China; Department of Radiation Oncology, Rush University Medical Center, Chicago, IL USA

**Keywords:** Prostate cancer, Radiotherapy, Seminal vesicle, Target delineation, CT reconstruction

## Abstract

**Background:**

Intermediate- to-high-risk prostate cancer can locally invade seminal vesicle (SV). It is recommended that anatomic proximal 1-cm to 2-cm SV be included in the clinical target volume (CTV) for definitive radiotherapy based on pathology studies. However, it remains unclear whether the pathology indicated SV extent is included into the CTV defined by current guidelines. The purpose of this study is to compare the volume of proximal SV included in CTV defined by EORTC prostate cancer radiotherapy guideline and RTOG0815 protocol with the actual anatomic volume.

**Methods:**

Radiotherapy planning CT images from 114 patients with intermediate- (36.8%) or high-risk (63.2%) prostate cancer were reconstructed with 1-mm-thick sections. The starting and ending points of SV and the cross sections of SV at 1-cm and 2-cm from the starting point were determined using 3D-view. Maximum (D_1H_, D_2H_) and minimum (D_1L_, D_2L_) vertical distance from these cross sections to the starting point were measured. Then, CTV of proximal SV defined by actual anatomy, EORTC guideline and RTOG0815 protocol were contoured and compared (paired t test).

**Results:**

Median length of D_1H_, D_1L_, D_2H_ and D_2L_ was 10.8 mm, 2.1 mm, 17.6 mm and 8.8 mm (95th percentile: 13.5mm, 5.0mm, 21.5mm and 13.5mm, respectively). For intermediate-risk patients, the proximal 1-cm SV CTV defined by EORTC guideline and RTOG0815 protocol inadequately included the anatomic proximal 1-cm SV in 62.3% (71/114) and 71.0% (81/114) cases, respectively. While for high-risk patients, the proximal 2-cm SV CTV defined by EORTC guideline inadequately included the anatomic proximal 2-cm SV in 17.5% (20/114) cases.

**Conclusions:**

SV involvement indicated by pathology studies was not completely included in the CTV defined by current guidelines. Delineation of proximal 1.4 cm and 2.2 cm SV in axial plane may be adequate to include the anatomic proximal 1-cm and 2-cm SV. However, part of SV may be over-contoured.

## Background

Localized prostate cancer can locally invade seminal vesicle (SV). The risk of SV involvement is usually more than 15-20% in patients with intermediate- and high-risk prostate cancer, according to detailed pathological studies [1-4]. Therefore, it is crucial to include at least the proximal SV in the clinical target volume (CTV) for definitive radiotherapy in these patients. Actually, the extent of SV involvement has already been investigated in patients with localized prostate cancer using prostatectomy specimens [5-8]. For example, Kestin *et al.* [6] reviewed 344 radical prostatectomy specimens, of which 81 demonstrated SV involvement. They found that the median length of SV involvement was 1-cm and the risk of involvement beyond 2-cm was less than 4% even in high-risk patients.

Based on pathological studies, inclusion of the proximal 1- to 2-cm SV in the CTV has been recommended for definitive radiotherapy of intermediate- and high-risk prostate cancer. However, the definition of proximal SV included in the CTV varies in many published guidelines. For example, the European Organization for Research and Treatment of Cancer (EORTC) recommends the proximal 2-cm SV along the vertical line included into the CTV in case of a high risk patient and the proximal 1-cm SV for intermediate-risk patients [9]. In the ongoing Radiation Therapy Oncology Group (RTOG) *Phase III Prospective Randomized Trial of Dose*-*Escalated Radiotherapy With or Without Short*-*term Androgen Deprivation Therapy for Patients With Intermediate*-*risk Prostate Cancer* (*RTOG0815*), only the proximal 1-cm of SV tissue adjacent to the prostate shall be included in the high-dose CTV. This 1-cm SV refers to both radial (in plane) and superior (out of plane) extent. Therefore it remains unknown that the difference in the volumes of proximal SV included in the CTV based on the above definitions compared with the anatomic proximal 1-cm or 2-cm SV and whether currently guidelines are adequate for inclusion of the SV involvement indicated by pathological studies.

In this study, we have contoured proximal 1-cm and 2-cm SV CTV according to the EORTC prostate cancer radiotherapy guidelines, RTOG0815 protocol and actual anatomy in the reconstructed CT images through 3-D view, and estimated the differences among the above three volumes. This study would potentially help determine the optimal proximal SV for definitive radiotherapy based on existing pathology knowledge about the tumor involvement into proximal SV in patients with intermediate-to-high prostate cancer.

## Methods

### Patients

Between April 2012 and December 2013, 134 consecutive patients with localized prostate cancer were treated with definitive intensity-modulated radiation therapy in Peking University First Hospital. All patients had a histological diagnosis of prostate adenocarcinoma from a trans-rectal biopsy. The study was approved by the Institutional Review Board of the Peking University First Hospital (No. 2013[656]).

### Imaging techniques

Radiotherapy planning CT images were obtained from a 16-slice CT simulator (Philips Brilliance Big Bore™, Philips, Amsterdam, Netherlands). Simulations were performed with the rectum as empty as possible and with a moderately full bladder (patients drinking approximately 500 ml water prior to simulation). Patients were scanned in the supine position from the superior aspect of L3 to 5 cm below the ischia tuberosity, and the images were reconstructed with a slice thickness of 1 mm.

### Delineation of anatomic proximal 1-/2-cm SV

Two experienced radiation oncologists processed and analyzed the CT images using the CT workstation (Philips Brilliance, Version: 3.5.4). After reconstruction, the workstation provided three orthogonal windows that were initially oriented in the sagittal, coronal, and axial planes. Then, the anatomic 1-/2-cm SV was delineated as follows.

#### Specification of the starting and ending points of the SV

**Figure 1:**
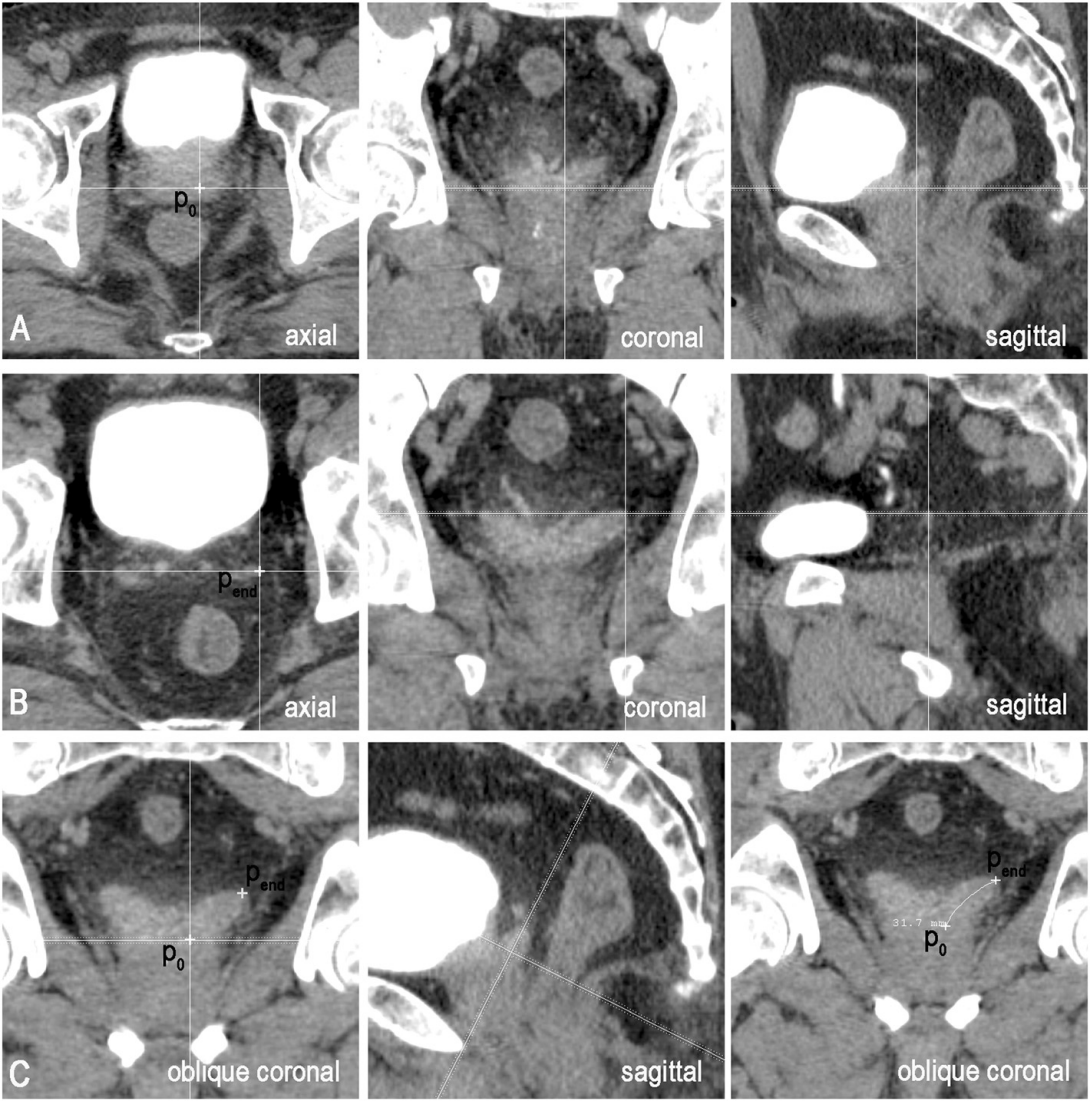
**Drawing the central line of SV. A.** P_0_ indicates the starting point of the SV, located in the first axial slice where both prostate and SV are visible. It is determined by referencing the coronal and the sagittal views to ensure that it is centered in the SV contour in both planes. **B.** P_end_ indicates the ending point of the SV in the last slice. The exact location needs to be determined by referencing the morphology in both the coronal and the sagittal planes. **C.** After locating the starting and ending points, an oblique coronal plane containing both endings is obtained by rotating the cut lines in the sagittal window. The central line is determined by drawing a curve connecting P_0_ and P_end_ along the middle line of the SV.

The starting point (P_0_, in Figure 1A) of each SV was located in the first axial plane where it appeared. The exact location was at the intersection of the SV central line with this plane, which was determined by referencing the coronal and sagittal views at the same time to ensure that P_0_ was centered in the SV contours in both these planes (Figure 1A). Move or rotate either window to see the morphology of the SV when necessary. The ending point (P_end_, in Figure 1B) of each SV was usually located in the last axial plane where it was visible. However, the exact location was determined by referencing the morphology of the SV in both the coronal and the sagittal planes (Figure 1B).

#### Drawing of the central line

After locating the starting and ending points, an oblique coronal plane (central line plane) containing both was obtained by rotating the cut lines in the sagittal window. The central line of the SV was delineated by drawing a curve connecting P_0_ and P_end_ along the middle line of the SV contour in this plane (Figure 1C). The rotation angle (α) of the coronal plane was defined as the posterior tilt angle of the SV.

#### Delineation of the anatomic 1-/2-cm SV on cut planes orthogonal to the central line

**Figure 2:**
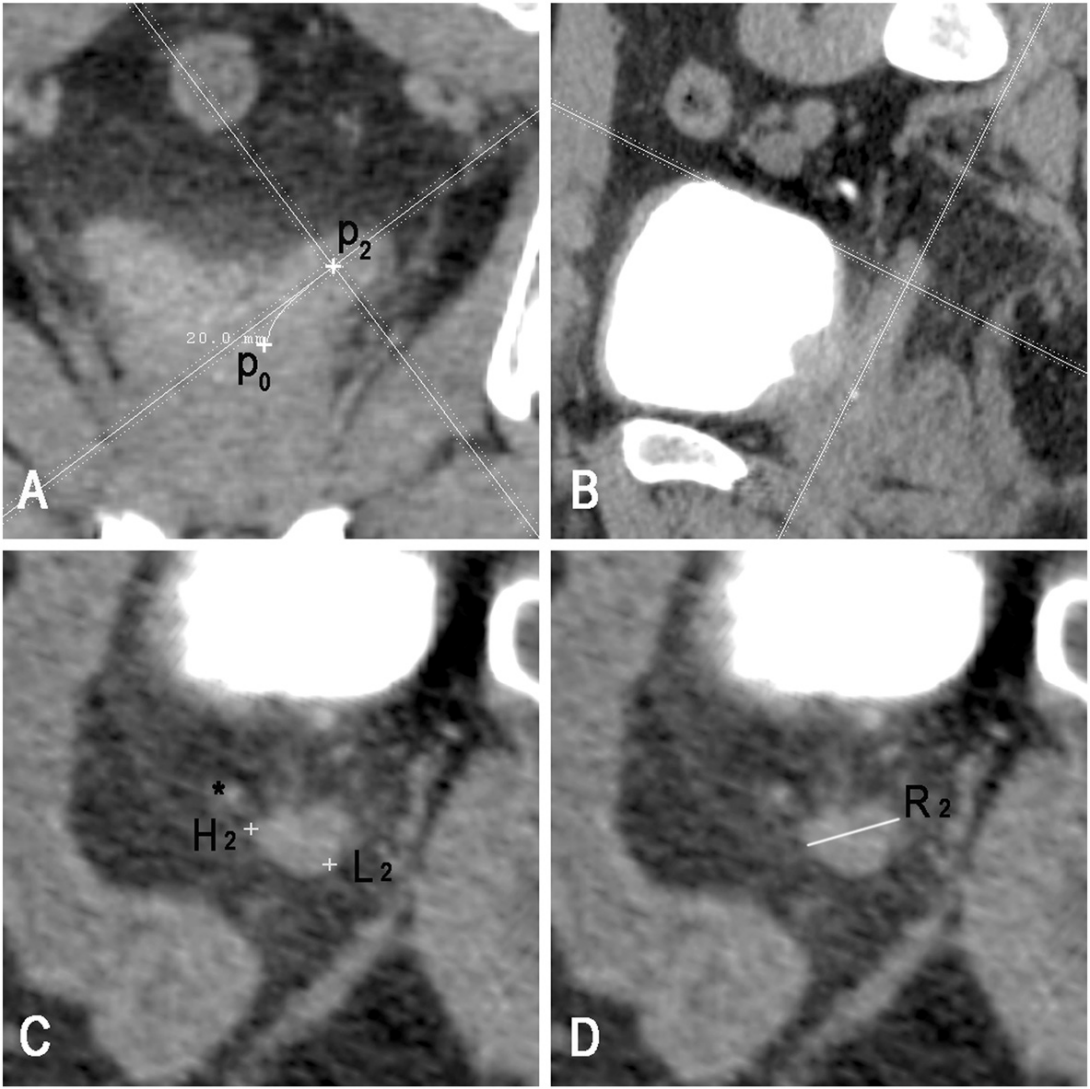
**Delineation of anatomic proximal 2** **-** **cm SV. A.** Locating a point 2-cm from the starting point of the SV on its central line (P_2_), cut lines in the oblique coronal plane are centered at P_2_ and adjusted until the sagittal cut plane becoming tangent to the central line. **B.** Oblique sagittal plane including the SV. **C.** Cut planes orthogonal to the central line. H_2_ and L_2_ indicate the points with maximum and minimum vertical distance to the starting plane of the SV. “*” indicates the vas deferens. **D.** Maximum diameter (R_2_) of the 2-cm cross section (R_2_).

We showed an example of delineating anatomic 2-cm SV. After locating a point 2-cm from the starting point of the SV on its central line (P_2_), cut lines in the oblique coronal plane were centered at P_2_ and adjusted until the sagittal cut plane becoming tangent to the central line (Figure 2A). At this point, the cross section of the SV was visualized in the axial window and the rotation angle of the sagittal plane (β) was defined as the lateral tilt angle of the SV (Figure 2C). Using the coordinate system provided by the CT workstation, the highest point (H_2_, Figure 2C) and lowest point (L_2_, Figure 2C) along the long axis of the body in this cross section were found and their vertical distances to the starting plane of the SV (D_2H_ and D_2L_) were recorded. The maximum diameter of this cross section (R_2_) was also recorded. The corresponding values for the 1-cm cross section were generated using a similar method.

### Volume of proximal SV CTV

**Figure 3:**
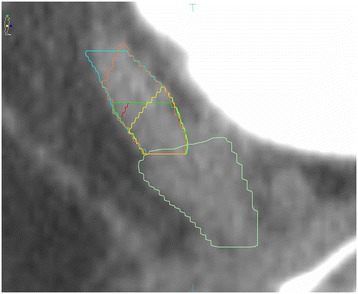
**Different volumes of proximal SV CTV.** Yellow line indicates V_ANAT-1_, red line indicates V_RTOG_, dark green line indicates V_EORTC-1_, orange line indicates V_ANAT-2_, blue line indicates V_EORTC-2_, and light green line indicates the prostate.

In order to obtain the volume of the SV CTV from the treatment planning system, two radiation oncologists with experience in prostate delineation were invited to participate in the study. Both observers were asked to delineate the SV CTV according to EORTC guideline, RTOG0815 protocol and actual anatomy, respectively (Figure 3). The proximal 1-cm or 2-cm SV CTV defined by EORTC guideline was contoured along the vertical line for 1-cm or 2-cm from the starting point. The proximal 1-cm SV CTV defined by RTOG was contoured to include the proximal 1-cm SV [both radial (in plane) and superior (out of plane)] adjacent to prostate gland. Anatomic 1-cm or 2-cm SV was contoured along the central line of SV from the starting point for 1-cm or 2-cm distance.

### Exclusion criteria

The feasibility of our method relies on the fact that few SVs show obvious curvature in the anteroposterior direction. Patients would be excluded from this study if the central line plane (oblique coronal plane) exceeded the middle third portion of the SVs, observing in the oblique sagittal window (Figure 2B).

### Statistical analysis

The Student’s paired and unpaired t test was used to determine the significance of the difference between two sample means. The association of characteristics with the CTV extent was analyzed using linear regression. Multivariate analyses were performed using multiple linear regression. A value of p < 0.05 was considered statistically significant. Statistical analysis was performed with SPSS version 19.0 (SPSS Inc., Chicago, IL, USA).

## Results

### Patients

According to the exclusion criteria, 114 patients were eligible for our study. The median age was 74 years (range: 54–82 years). Based upon current National Comprehensive Cancer Network prognostic risk groupings, 42 patients (36.8%) were categorized into an intermediate-risk group and 72 patients (63.2%) into a high-risk group.

### Anatomic characteristics of SV

For the entire cohort, the median length of the SV was 36.7 mm (mean: 36.4 mm, range: 21.5–55.8 mm). The posterior tilt angle of the SV (α) was 29.6 ± 12.9°, and the lateral tilt angle (β) was 38.4 ± 7.9°. The maximum diameter of the 1-cm and 2-cm cross section was 16.6 ± 3.6 mm and 15.9 ± 3.7 mm, respectively.

### Extent of anatomic proximal 1-/2-cm SV

The median length of D_1H_, D_1L_, D_2H_ and D_2L_ was 10.8 mm (95th percentile: 13.5 mm), 2.1 mm (95th percentile: 5.0 mm), 17.6 mm (95th percentile: 21.5) and 8.8 mm (95th percentile: 13.5 mm), respectively (Table 1). All the D_1H_ and D_2H_ located in the anterior or inner portion of the SV, while all the D_1L_ and D_2L_ located in posterior or lateral portion of the SV (Figure 2C).

**Table 1:** **Extent of anatomic proximal 1**-/**2**-**cm SV**

	**Mean** ± **SD**	**Median**	**95th percentile**	**Range**
*D*_1H_ (mm)	10.6 ± 1.8	10.8	13.5	6.0 – 14.1
*D*_1L_ (mm)	2.1 ± 2.0	2.1	5.0	-3.1 – 7.0
*D*_2H_ (mm)	17.2 ± 2.9	17.6	21.5	9.0 – 24.6
*D*_2L_ (mm)	8.8 ± 2.7	8.8	13.5	3.5 – 14.7

*Abbreviations*: *D*_*1/2H*_ = maximum vertical distance from the 1-/2-cm cross section to the starting plane of the SV; *D*_*1/2L*_ = minimum vertical distance from the 1-/2-cm cross section to the starting plane of the SV.

According to our measurement, for intermediate-risk prostate cancer, the proximal 1-cm SV CTV defined by RTOG0815 and EORTC guideline was inadequate for inclusion of anatomic proximal 1-cm SV in 62.3% (71/114) and 71.0% (81/114) of cases, respectively. While for high-risk prostate cancer, the proximal 2-cm SV CTV defined by EORTC guideline was inadequate for including anatomic proximal 2-cm SV in 17.5% (20/114) of cases. All of SV portion missed by EORTC guideline and 91.3% of that missed by the RTOG protocol located in the anterior or inner portion of the SV (Figure 3).

### Factors affecting the extent of anatomic proximal SV

On univariate analysis, a smaller posterior tilt angle of the SV (α) and a larger diameter of the cross section (R_1/2_) were associated with a larger D_1H_ or D_2H_ ( p < 0.01). On multivariate analysis, posterior tilt angle (α) and maximum diameter (R_1/2_) remained significantly associated with length of D_1H_ and D_2H_. Additionally, a smaller lateral tilt angle (β) of the SV was significantly associated with a longer D_2H_ (p < 0.01).

### Volumes of proximal SV CTV defined by different standards

We compared volumes of the proximal SV CTV defined by actual anatomy (V_ANAT-1/2_), RTOG0815 protocol (V_RTOG_) and EORTC guideline (V_EORTC-1/2_). For intermediate-risk prostate cancer, the average volume of V_ANAT-1_, V_RTOG_ and V_EORTC-1_ was 3.66 ± 0.92 cm^3^, 4.09 ± 0.92 cm^3^ and 4.80 ± 1.18 cm^3^, respectively. For high-risk prostate cancer, the average volume of V_ANAT-2_ and V_EORTC-2_ was 6.23 ± 1.94 cm^3^ and 8.67 ± 2.29 cm^3^, respectively. The volume differences between V_ANAT-1_, V_RTOG_ and V_EORTC-1_, and between V_ANAT-2_ and V_EORTC-2_ were all significant (paired t tests, all p < 0.001; Table 2).

**Table 2:** **Volumes of proximal SV CTV defined by different standards**

**SV**-**volume** **(** **cm** ^**3**^ **)**	**Mean** **±** **SD**	**Median**	**Range**
V_ANAT-1_	3.66 ± 0.92*	3.69	1.65 – 5.28
V_RTOG_	4.09 ± 0.92*	4.18	2.67 – 6.44
V_EORTC-1_	4.80 ± 1.18*	4.72	2.89 – 7.41
V_ANAT-2_	6.23 ± 1.94 ^y^	6.25	2.68 – 12.54
V_EORTC-2_	8.67 ± 2.29 ^y^	8.60	4.71 – 14.96

*Abbreviations*: *V*_*ANAT-1/2*_ = volume of proximal 1-/2-cm SV CTV defined by actual anatomy; *V*_*RTOG*_ = volume of proximal 1-cm SV CTV defined by RTOG0815; *V*_*EORTC-1/2*_ = volume of proximal 1-/2-cm SV CTV defined by EORTC guideline; *p < 0.001 for comparison between any two means of V_ANAT-1_, V_RTOG_ and V_EORTC-1_; ^y^p < 0.001 for the comparison between V_ANAT-2_ and V_EORTC-2_.

## Discussion

SV involvement was indicated in more than 15-20% of intermediate- to-high-risk prostate cancer during post-prostatectomy pathological examinations [1-4]. Since numbers of studies have already shown that SV involvement is associated with higher rates of biochemical recurrence, metastasis and reduced overall survival, it is advisable to include proximal SV in the CTV for definitive radiotherapy of patients with intermediate- to-high-risk prostate cancer [10,11].

Unfortunately, there is no agreed-upon standard of the optimal extent of the proximal SV should be included within the CTV in these patients. Numbers of studies have focused on this issue. Kestin *et al*. [6] performed by far the most detailed analysis of the extent of SV involvement in localized prostate cancer. By reviewing 344 prostatectomy samples, they found the median length of SV involvement was 1.0 cm and only 1% of their patients had a risk of SV involvement beyond 2.0 cm. Furthermore this risk was less than 4% even in high-risk patients. With a complete SV resection rate of 85%, Kestin and his colleagues were confident that only the proximal 2.0- to 2.5-cm of the SV should be included within the CTV for definitive radiotherapy for localized prostate cancer. Other studies came to similar conclusions. Though there were differences in terms of the percentile of involvement, all existing pathological studies reached an agreement that SV involvement beyond the proximal 2.0 cm was very rare [5,7,8,12].

Based on these findings, EORTC prostate cancer radiotherapy guideline recommends that proximal 2-cm SV be included in the CTV for high-risk prostate cancer, and proximal 1-cm SV for intermediate-risk cases [9]. While, in the ongoing RTOG0815 protocol for intermediate-risk patients, the proximal 1-cm of SV tissue adjacent to the prostate is recommended to be irradiated and this 1-cm refers to both radial (in plane) and superior (out of plane) extent. It is worth noting that CTV defined by these guidelines are delineated in axial plane CT images. While, SV extends in a posterlateral direction but not orthogonal to the axial planes. So, there may be differences in proximal SV defined by the present guidelines compared with that defined by actual anatomy. However, no study has ever concerned this issue.

In this study, with help of reconstructed thin-slice CT images, we were able to locate the anatomic proximal 1-cm and 2-cm SV. Extent of proximal 1-cm or 2-cm SV defined by actual anatomy, RTOG0815 and EORTC guideline were contoured and compared in 114 patients. Although, volume of proximal SV defined by current guidelines was much larger than that defined by actual anatomy, part of SV involvement indicated by pathological studies may be missed. According to our measurements, maximum vertical distance from the 1-cm and 2-cm cross section to the starting point of the SV exceeded 1.0 cm and 2.0 cm in 62.3% and 17.5% of patients, respectively. These exceeded SV portions accounted for all of the anatomic proximal 1-cm or 2-cm SV missed by CTV defined by EORTC guideline and for 91.3% percent of that missed by CTV defined by RTOG protocol. It is therefore advisable to raise the upper margin of the CTV, maybe to 1.4 cm from the starting plane for intermediate-risk patients and to 2.2 cm for high-risk patients.

**Figure 4:**
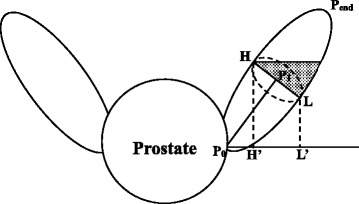
**Relationship between CTV extent and anatomic SV included.** Schematic diagram indicates the cross section of the SV. P_0_P_end_ indicates the central line. HH’ and LL’ indicate the maximum and minimum vertical distance to the starting plane of the SV. Dotted area indicates the part of SV that might be over-contoured for irradiation.

It is also worth noting that the extent of proximal SV included in the CTV defined by current guideline is much larger than desired. Here we want to use a schematic diagram to explain this issue (Figure 4). To include the anatomic proximal 1-cm and 2-cm SV, it is necessary to include the highest point in the 1-cm and 2-cm cross section of the SV (H, Figure 4). This is because the SV goes in an oblique direction and has a certain volume. Maximum diameter of the 1-cm and 2-cm cross section was 16.6 ± 3.6 mm and 15.9 ± 3.7 mm according to our measurements. However, part of SV tissue beyond the proximal 1-cm or 2-cm SV would also be irradiated (dot area in Figure 4). The minimum vertical distance from the starting point to the 1-cm and 2-cm cross sections (D_1L_ and D_2L_) were measured to evaluate the volume of SV over-contoured. The median length of D_1L_ and D_2L_ was 2.1 mm (95th percentile: 5.0 mm) and 8.8 mm (95th percentile: 13.5 mm), respectively. In addition, we found that all of the highest points of the 1-cm and 2-cm cross sections located in the anterior or inner part of the SV, while all of the lowest points located in posterior or lateral part of the SV.

Taking all these founding together, we are confident that delineating the proximal 1.4 cm and 2.2 cm SV in the axial plane could entirely include the anatomic proximal1-cm and 2-cm SV in at least 95% of cases, respectively. Meanwhile, part of SV tissue beyond this extent would also be include in the CTV, which may increase the volume of radiated normal tissues and hinder dose escalation. Therefore, partial of the posterior and lateral SV tissue between the upper and lower margin of the 1-cm (0.5 to 1.4 cm) and 2-cm (1.4 to 2.2 cm) cross section would be excluded from the CTV. However, this should be done carefully as anatomic proximal 1-cm or 2-cm SV can not be located or referenced in axial planning CT images.

## Conclusions

Though, volume of proximal SV included in the CTV defined by published guidelines is much larger than the volume of anatomic 1-cm and 2-cm SV, part of SV involvement indicated by pathology studies may be missed. For intermediate- and high-risk patients, delineation of proximal 1.4 cm and 2.2 cm SV in axial plane may be adequate to include the anatomic proximal 1-cm and 2-cm SV respectively. However, part of posterior and lateral portion (from 0.5 to 1.4 cm for intermediate-risk cancer, from 1.4 to 2.2 cm for high-risk patients) of SV within this extent may be over-contoured for irradiation.

## Abbreviations

SV: Seminal vesicles
CTV: Clinical target volume
CT: Computed tomography
EORTC: European Organization for Research and Treatment of Cancer
RTOG: Radiation Therapy Oncology Group
